# 

**Published:** 1909-12

**Authors:** 


					CONTENTS.
Page
The History and Practice of Surgery in Ancient and Mediaeval
Times.
By James Swain, M.S., M.D. Lond., F.R.C.S. Eng.
289
The Mercurial Treatment of Tabes Dorsalis.
By Maurice
Faure, M.D.
312
" Vis Medicatrix Naturae."
By G. Munro Smith, M.R.C.S.
321
Cases of Pneumonia.
By Arthur Fells, M.B., C.M. Edin. ...
336
Progress of the Medical Sciences :
Medicine.
George Parker, M.D., M.A. Cantab.
339
Obstetrics.
D. C. Rayner, F.R.C.S.
343
Surgery.
H. G. Kyle, M.B., B.Ch. Oxon.
346
Reviews of Books
349
Editorial Notes
368
Notes on Preparations for the Sick
374
The Library of the Bristol Medico-Chirurgical Society
377
Meetings of Societies
378
Local Medical Notes
383

				

